# Supplementary material for: Comparative Proteomic Analysis Identifies EphA2 as a Specific Cell Surface Marker for Wharton’s Jelly-Derived Mesenchymal Stem Cells
Source: Int J Mol Sci. 2020 Sep 3;21(17):6437. doi: 10.3390/ijms21176437 (PMC7503404; doi:10.3390/ijms21176437)
Supplement: Supplementary file 1 [file ijms-21-06437-s001.pdf]

Comparative proteomic analysis identifies EphA2 as a specific cell surface marker for Wharton's Jelly-derived mesenchymal stem cells

Ashraf Al Madhoun [1,2,*], Sulaiman K. Marafie [3], Dania Haddad [2], Motasem Melhem [2], Mohamed Abu-Farha [3], Hamad Ali [2,4], Sardar Sindhu [1], Maher Atari [5] and Fahd Al-Mulla [2]

Supplementary Table 1: Antibodies used in the study.

Antibody	Catalogue number	Manufacturer
EPHA2	AF3035	R&D
SLC25A4	ab102032	Abcam
TALIN2	NBP2-50322	NOVUSBIO
CLE7	ab188326	abcam
LMF2	NBP-159374	NOVUSBIO
CD49b	1233	R&D
VDAC3	AB130561	ABCAM
SOD2	AB16956	ABCAM
PLEC	SAB1404217	Sigma-Aldrich
NEXILIN	AB213628	ABCAM
CDH5	ab33168	ABCAM
CD49e	**MAB1864**	NOVUSBIO
CDH2	14215S	CELL SIGNALLING
MLCK1	NBP1-87744	NOVUSBIO
Actin Ab-5	612657	BD Pharmingen

Supplementary Table 2: Primers used in the study.

Gene	Forward primer (5'-3')	Reverse primer (5'-3')
CLE7	CCTGCTTCAGATTCAGCGTC	AAGCAACAGGTAAGCCCTCT
PLEC3	ACGAGATCAGCTCCCTCAAAG	CCATCGCGGAGGTCTTCATAC
IGF2BP3	CTGCACGGGAAACCCATAGA	CCAGCACCTCCCACTGTAAAT
Talin	ACGATGCGTGTCGAGTCATT	AGCCAAATCCCTTTCCTCGG
LMF2-F1	TCATCTACTTGCTGCTTTGGG	AAGCCAGTCTCTAGCAGCAG
Ncadherin	GAAATGTCTCCTGTGGGTGC	GGTTGTCCCTGAAGAATGCTG
EphA2-F	GTGCAGTGGATGGCGAGT	GGCTCTCAGATGCCTCAAAC
CD49e	CCTTCTTCGGATTCTCAGTGG	CCAAGGACAGAGGTAGACA
Nexilin	AGAGGAAGAAAAGAAGGCGTT	CCAGTTTTCCCGGTGTAAGAAAT
SOD2	GTCACCGAGGAGAAGTACCA	TTGATATGACCACCACCATTGA
CD49b	CCCAATATGGTGGGGACCTC	CCCACCAGAAGCTGCTGAATA
VDAC3	GGAAAGGCTGCTAAGGATGTC	AAATTCCACTCCACTACAAGACT
SLC25A4	ATCGAGAGGGTCAAACTGCT	CTTAGGGATTCTCACCACACA
CDH5	ATGAGATCGTGGTGGAAGCG	TGTGTACTTGGTCTGGGTGA
CD73	GCCTGGGAGCTTACGATTTTG	TAGTGCCCTGGTACTGGTCG
